# 284. Post-Acute Sequelae of COVID-19 Two Years After Acute Infection

**DOI:** 10.1093/ofid/ofac492.362

**Published:** 2022-12-15

**Authors:** Jennifer Logue, Nicholas M Franko, Megan M Kemp, Denise J McCulloch, Eric J Chow, Helen Y Chu

**Affiliations:** University of Washington, Seattle, Washington; University of Washington, Seattle, Washington; University of Washington, Seattle, Washington; University of Washington, Seattle, Washington; Public Health - Seattle & King County, Seattle, Washington; University of Washington, Seattle, Washington

## Abstract

**Background:**

The post-acute sequelae of COVID-19 (PASC) includes a constellation of debilitating symptoms after SARS-CoV-2 infection. Much remains unknown about the long-term health burden of COVID-19. We describe the symptom course and quality of life of adults up to 2 years after mild acute COVID-19.

**Methods:**

Adults within 30 days of laboratory-confirmed acute COVID-19 were enrolled as cases from January – September 2020 and followed for 2 years. Demographic and symptom data were collected in an enrollment survey and at 6, 12 and 24 months post-infection. Surveys included vaccination status, symptom course, and quality of life assessments (Fatigue Assessment Scale (FAS) and EuroQual visual analog scale (VAS)). A cohort of SARS-CoV-2 uninfected controls was concurrently enrolled and surveyed. We used descriptive statistics to compare cases and controls and defined a p-value < 0.05 as significant.

**Results:**

A total of 112 of 239 enrolled cases and 44 of 59 controls completed all surveys. Of the 112 cases, 105 (94%) had mild disease. In the 6, 12 and 24 month surveys, 39 (35%), 48 (43%) and 56 (50%) cases indicated at least one persistent symptom, respectively, compared to 4 (9%), 5 (11%) and 6 (14%) controls (Table 1). In all 3 surveys, fatigue and altered smell or taste were the most common post-infection symptoms among cases (Figure 1). At 2 years, 40 (36%) cases reported symptoms were improving or resolved and 30 (27%) reported symptoms continued to wax and wane. Symptoms improved and worsened for 10 and 4 cases, respectively, following a complete SARS-CoV-2 vaccination. 46% reported seeking medical attention for persistent symptoms and 34% of those employed reported symptoms negatively impacted their ability to work. When compared to controls in the 12 and 24 month surveys, cases had a significantly higher mean FAS score (p-value < 0.001 and 0.01, respectively) and significantly lower VAS score (p-value = 0.01 and < 0.001, respectively).

Demographic and Clinical Characteristics of the Study Cohort

**Figure:**
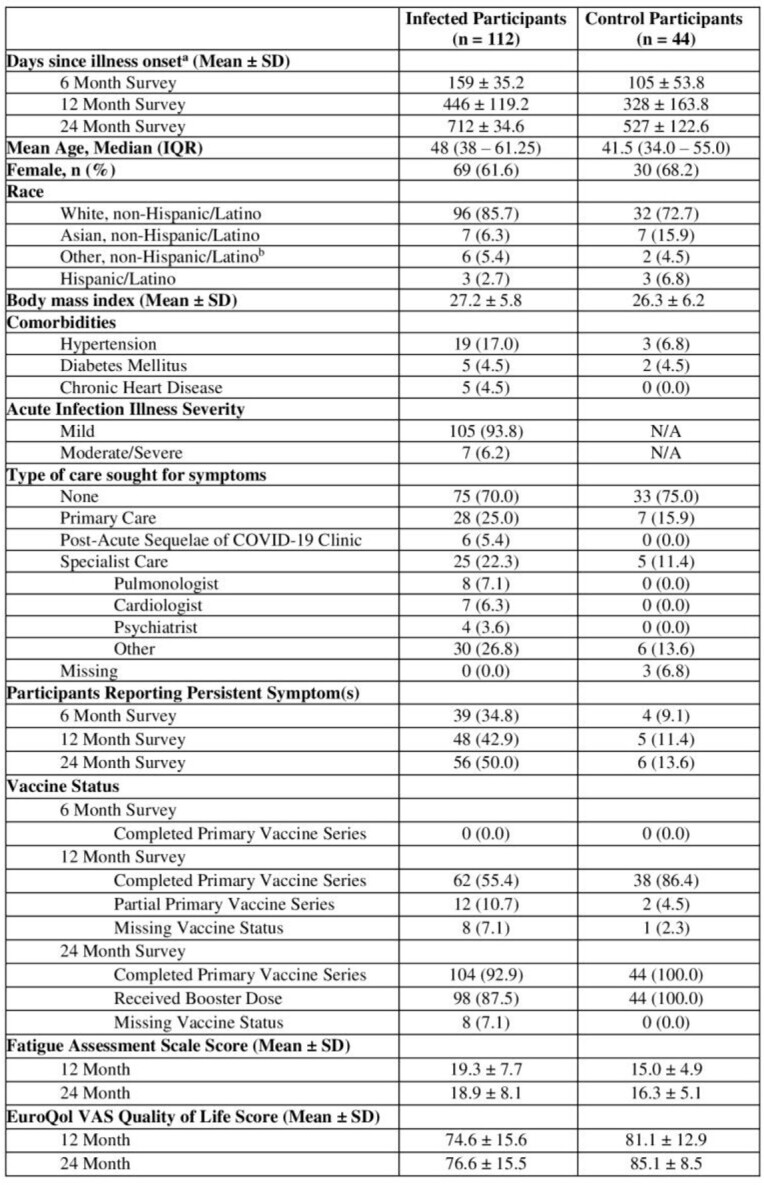

a. Time since symptom onset in infected cohort and time since enrollment in healthy controls

b. Other race/ethnicity included American Indian or Alaska Native, Black or African American, Native Hawaiian or other Pacific Islander, and more than 1 race.

Percentage of participants reporting symptoms at 6-, 12-, and 24-months

**Figure:**
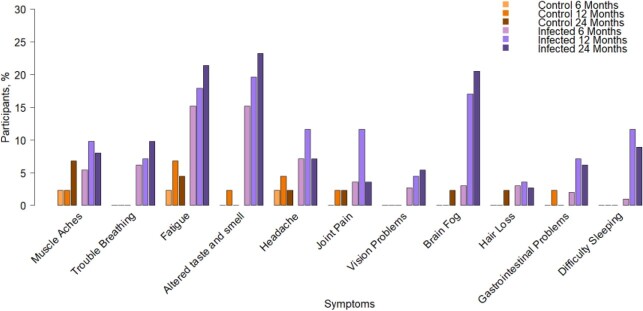

*

**Conclusion:**

Symptoms associated with PASC were reported up to 2 years after infection with significant impacts on quality of life. These findings underscore the healthcare and societal burdens even after recovery from acute infection. As studies seek to identify the underlying mechanisms of PASC, prevention of acute infection remains the mainstay of COVID-19 burden mitigation.

**Disclosures:**

**Helen Y. Chu, MD, MPH**, Cepheid: Reagents|Ellume: Advisor/Consultant|Gates Ventures: Grant/Research Support|Merck: Advisor/Consultant|Pfizer: Advisor/Consultant.

